# Mycorrhiza-Induced Resistance against Foliar Pathogens Is Uncoupled of Nutritional Effects under Different Light Intensities

**DOI:** 10.3390/jof7060402

**Published:** 2021-05-21

**Authors:** Judith Pozo de la Hoz, Javier Rivero, Concepción Azcón-Aguilar, Miguel Urrestarazu, María J. Pozo

**Affiliations:** 1 Department of Agronomy, University of Almería, Ctra. Sacramento s/n, 04120 Almería, Spain; judith.pozo@eez.csic.es (J.P.d.l.H.); mgavilan@ual.es (M.U.); 2 Department of Soil Microbiology and Symbiotic Systems, Estación Experimental del Zaidín, CSIC, Profesor Albareda 1, 18008 Granada, Spain; javier.rivero@eez.csic.es (J.R.); cazcon@eez.csic.es (C.A.-A.)

**Keywords:** *Botrytis cinerea*, light intensity, arbuscular mycorrhiza, mycorrhiza-induced resistance, plant growth, plant nutrition

## Abstract

The use of microbial inoculants, particularly arbuscular mycorrhizal fungi, has great potential for sustainable crop management, which aims to reduce the use of chemical fertilizers and pesticides. However, one of the major challenges of their use in agriculture is the variability of the inoculation effects in the field, partly because of the varying environmental conditions. Light intensity and quality affect plant growth and defense, but little is known about their impacts on the benefits of mycorrhizal symbioses. We tested the effects of five different light intensities on plant nutrition and resistance to the necrotrophic foliar pathogen *Botrytis cinerea* in mycorrhizal and non-mycorrhizal lettuce plants. Our results evidence that mycorrhiza establishment is strongly influenced by light intensity, both regarding the extension of root colonization and the abundance of fungal vesicles within the roots. Light intensity also had significant effects on plant growth, nutrient content, and resistance to the pathogen. The effect of the mycorrhizal symbiosis on plant growth and nutrient content depended on the light intensity, and mycorrhiza efficiently reduced disease incidence and severity under all light intensities. Thus, mycorrhiza-induced resistance can be uncoupled from mycorrhizal effects on plant nutrition. Therefore, mycorrhizal symbioses can be beneficial by providing biotic stress protection even in the absence of nutritional or growth benefits.

## 1. Introduction

Current agriculture demands safe and environmentally friendly strategies to reduce the use of chemical fertilizers and pesticides. In this context, the use of microbial inoculants increasing in popularity due to their beneficial effects on plant growth and performance [1]. Beneficial plant–microbe interactions are widespread in nature, and their potential benefits for plant growth and health are well documented [2,3,4]. Some of the mechanisms underlying such benefits have been unveiled, usually under controlled lab conditions [1,5,6]. Despite the high interest of the industry in the biotechnological applications of beneficial microbes in agriculture (e.g., http://www.biostimulants.eu/ accessed on 10 December 2020), the application of microbial inoculants is still challenging due to the unpredictability of the results when applied under field or real production conditions. The outcome of the plant–microbe interaction depends on the genotype of each partner, the biotic context (i.e., the microbial communities present in the rhizosphere where inoculants are applied), and the environmental conditions [3,7]. Thus, understanding the “context dependency” of plant–microbe interactions is essential in order to optimize biotechnological applications of microbial inoculants [7,8,9].

Among soil-benefiting microbes, arbuscular mycorrhizal fungi (AMF) are of utmost ecological importance and have great potential for sustainable crop management [4,10,11]. AMF establish intimate mutualistic associations, known as arbuscular mycorrhizas (AM), with the roots of more than 70% of terrestrial plant species, including most vegetable crops [12]. The symbiosis is mostly known for the capacity of the fungus to improve the acquisition of water and mineral nutrients—mainly phosphorus (P)—by the plant [13,14,15], but AMF can also improve plant tolerance to multiple stresses. In fact, AM enhances plant phenotypic plasticity, an important advantage in heterogeneous and changing environments where the precise allocation of limited resources between growth and stress resistance is critical for survival [15,16,17]. Mycorrhizal plants are usually more resistant to abiotic stresses such as drought, salinity, and heavy metal pollution [17,18,19], but AMF can also increase plant resistance/tolerance to biotic stresses by triggering the so-called mycorrhiza-induced resistance (MIR) against diverse root and foliar pathogens and pests [20,21,22,23,24,25,26,27,28]. In return, AMF require photosynthates and lipids from the plant to complete their life cycle. AMF colonize the root cortex inter and/or intracellularly, forming highly branched structures called arbuscules in which the exchange of nutrients takes place. The fungi deliver to the root cells P acquired by their network of extraradical mycelia from soil areas hardly accessible to roots, and other nutritional benefits have been reported, including improved iron, zinc, copper, sulfur, and nitrogen acquisition [15,29]. In return, it has been estimated that up to 20% of plant-fixed carbon compounds can be transferred to the AMF [30] constituting the “symbiotic costs.” A delicate balance between the symbiosis costs and benefits rules the interaction, and although growth promotion is common in mycorrhizal plants, negative mycorrhizal growth responses (MGR) have also been observed in certain plant–AMF combinations or environmental conditions [31,32,33,34,35]. It is accepted that plants control the extent of fungal colonization according to the environmental conditions and their needs [16]. It has been reported that in plants colonized by multiple AMF, plant preferential allocation of resources towards the most beneficial mutualist depends upon above-ground resources [36]. Thus, understanding the regulation of AM symbiosis and its benefits under different conditions is required to understand its context-dependency [16,33,35,37].

Light is one of the key factors affecting plant performance. It directly influences plant growth and development [38,39]. Light perception by plant photoreceptors determines important programs such as photomorphogenesis and shade avoidance, and regulates plant defenses [40]. Experimental evidence reveals that abiotic factors such as light, but also others, such as circadian rhythm and temperature, have regulatory roles in plant immunity and induced resistance [41,42,43]. Some induced defenses are actively repressed when the plant strives for light in a canopy: there is a trade-off where the fight for light is prioritized over the fight against potential aggressors. Thus, light can be considered a positive modulator of plant defenses [40,43]. Besides the effects on plant growth and defense, light may also have a direct impact on the microbes [44,45,46,47]. Although a considerable impact of light on plant–microbe interactions is to be expected, this has been poorly explored [48,49]. Several studies have addressed the impact of light intensity on mycorrhizal establishment, usually relating low light intensities with reductions of mycorrhizal colonization [34,35,50]. Light intensity has also been proposed to underlie the variability of the plant growth response to mycorrhizal colonization in different systems [34,51]. However, little is known about light’s effects on mycorrhiza-related stress tolerance.

To investigate the effects of light intensity on mycorrhizal symbiosis establishment and its impacts on plant growth, nutrition, and MIR, we selected the lettuce *Botrytis cinerea* pathosystem. *Lactuca sativa* L. is considered the most important vegetable in the group of leafy vegetables. In 2019, over 29 million tons of lettuce were produced worldwide [52]. Grey mold caused by the necrotrophic fungus *Botrytis cinerea* has been considered the major disease in greenhouse-grown lettuce. *B. cinerea* is one of the most important plant pathogens because of its broad host range and its ability to cause severe damage both pre- and post-harvest [53]. Under favorable conditions, *Botrytis* conidia in the soil and in infected plant debris rapidly germinates and can colonize lettuce stems and leaves [54]. Remarkably, mycorrhizal colonization has been shown to reduce *Botrytis* proliferation and damage in tomatoes, another relevant crop [23,25,26,55]. In previous studies, mycorrhizal colonization was shown to improve lettuce nutritional content and enhanced abiotic stress tolerance [18,56,57,58,59], but some of those benefits depended on the crop management or conditions [56,57]. The potential bioprotective effects of the mycorrhizal symbiosis on lettuce remain unexplored.

The aim of this research was to explore the effects of light intensity on mycorrhizal establishment and functioning in lettuce plants, and the effects of the symbiosis on plant fitness. We hypothesized that light intensity impacts mycorrhizal root colonization, and has effects on host nutrient contents, growth response, and resistance against foliar pathogens. Our results show that while light strongly impacts AMF colonization and modulates the symbiosis effects on plant growth and nutrition, the symbiosis reduced disease incidence and severity under all light intensities. 

## 2. Materials and Methods

### 2.1. Biological Material and AMF Inoculation 

The arbuscular mycorrhizal fungus *Rhizoglomus irregulare* (DAOM197198, Syn. *Glomus intraradices*) was obtained from the Estación Experimental del Zaidín AMF germplasm collection, and maintained as a sand-vermiculite based inocula in an open-pot culture of *Trifolium repens* L. A mix of the substrate, spores, mycelia, and infected root fragments from these cultures were used as inocula [60].

Lettuce seeds (*Lactuca sativa* L. cv. “A foglia di quercia”) were surface-sterilized in commercial bleach 10% (*v*/*v*) and rinsed thoroughly with sterile water. Seeds were sown in plastic seedling trays 25 mL cells containing a volcanic rock:coconut fibers (1:1) mixture. For the mycorrhizal treatments, *R. irregulare* inoculum was added to the growing substrate (50%, *v*/*v*). Uninoculated control plants received the same amounts of sterile sand-vermiculite substrate. The planting density was 144 plants per m^2^.

### 2.2. Experimental Design and Growing Conditions

A total of six plants were used for each treatment. The experiment consisted of a randomized design with two inoculation treatments: (1) non-inoculated control plants (non-mycorrhizal, Nm) and (2) plants inoculated with the AM fungus *R. irregulare* (Ri); and five light intensity treatments: I1, I2, I3, I4, and I5.

**Figure 1:**
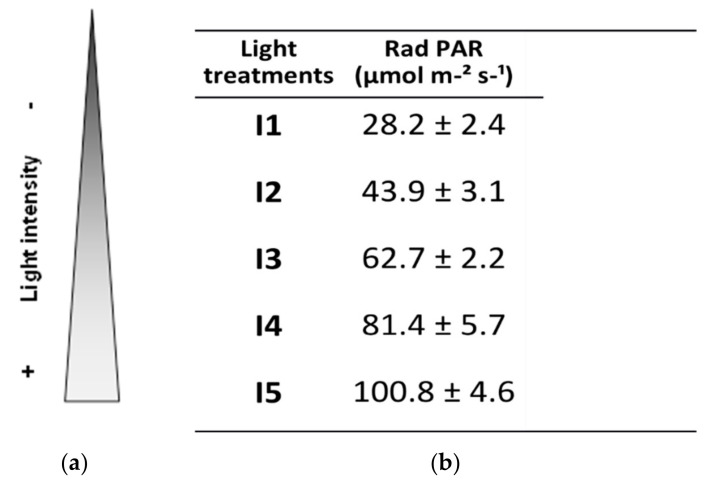
Light treatments. Plants grew on 5 different shelves with 2, 3, 4, 5, and 6 LED tubes that refer to treatments I1, I2, I3, I4, and I5, respectively. The resulting light intensity for each treatment is presented, and expressed as photosynthetically active radiation (PAR) (mol µm^−2^ s^−1^).

Seedling trays were placed randomly in a vertical phytotron compartmented into 5 shelves, I1, I2, I3, I4, and I5 (Figure 1), with 2, 3, 4, 5, and 6 Roblan^®^ LED tubes respectively. The characteristics of the tubes are detailed in Table 1.

Photosynthetically active radiation (PAR) per light intensity level was determined experimentally as photon photosynthetic flux density (PPFD) with ADC BioScientific Ltd.’s LCi Portable Photosynthesis System (BioScientific Ltd, Hoddesdon, UK). Accordingly, the light intensity treatments are defined in Figure 1. 

A 16/8 h diurnal photoperiod and 50–70% humidity were applied, and temperature ranged from 25 to 32 °C during the day and 15 to 22 °C by night. Plants were watered at 100% field capacity every other day with ½ Long Ashton nutrient solution [61] containing 25% of the standard phosphorus (P) concentration. Three weeks later, plants with three true leaves were transplanted into 500 mL pots containing volcanic rock as a substrate [62]. For each light treatment, when 50% of the easily available water in the substrate was depleted, plants were watered until reaching 15% to 25% drainage [63].

### 2.3. Plant Harvesting and Determinations

Plants were harvested after 7 weeks of growth. Shoot and root fresh weights were determined and an aliquot of each root system was reserved for mycorrhizal evaluation. One leaf per plant was harvested for the pathogen bioassays and immediately inoculated with *B. cinerea*, as described below. The rest of the plant was oven-dried at 70 °C for 72 h and used for dry weight determination. Dry shoots were then ground to powder and used for nutrient analysis. 

Mycorrhizal colonization was estimated after clearing washed roots in 10% KOH and subsequent staining of fungal structures with 5% ink in 2% acetic acid, according to Vierheilig and coworkers [64]. Mycorrhizal colonization, expressed as a percentage of total root length colonized by the AMF, was calculated according to the gridline intersect method [65], using a Nikon Eclipse 50i dissecting microscope (Nikon, Tokyo, Japan). A more detailed analysis of mycorrhizal structures was performed at higher magnification with a compound microscope: Mycorrhizal frequency (F%), mycorrhizal intensity (M%), mycorrhizal intensity of colonized root fragments (m%), arbuscular richness (A%), arbuscular richness of colonized root fragments (a%), vesicle richness (V%), and vesicle richness of colonized root fragments (v%) were assessed according to Trouvelot et al. [66].

### 2.4. Botrytis Cinerea Inoculation and Disease Assessment

*Botrytis cinerea* was grown in potato dextrose agar plates, supplemented with freeze-dried tomato leaves. Plant inoculation was performed on a detached leaf per plant with 5-mm-diameter agar plugs containing actively growing hyphae of *B. cinerea* from 3-week-old cultures. Inoculated leaves were incubated at high humidity in plastic trays covered with polyethylene wrap at 22 °C in the dark. Disease incidence (percentage of inoculated leaves with symptoms) and disease severity were determined for each treatment. A 0–3 disease index scale was used: 0, no visible symptoms; 1, necrotic lesions extending below 25% of the leaf surface; 2, necrosis extending more than 50% of the leaf surface; 3: dead leaf.

### 2.5. Mineral Nutrients Analyses in Plant Tissues

Mineral composition in leaves was measured at the Ionomic Laboratory of the Technical Services of the Estación Experimental del Zaidin (EEZ-CSIC) in Granada, Spain. Three independent biological replicates, each consisting of a pool of two plants, were analyzed for each treatment. Element concentrations were analyzed after acid digestion of the samples, by inductively coupled plasma optical emission spectrometry (ICP-OES; ICAP 6500 Duo Thermo, ThermoScientific, Waltham, MA, USA). 

### 2.6. Statistical Analyses

Data were subjected to ANOVA Multifactorial analyses (*p* < 0.05), and where appropriate, means were compared by the LSD posthoc test. The software packages used were Statgraphics Centurion^®^ 16.1.15 and Microsoft Office 2010. Regarding nutrient content, global signal behavior was determined by principal component analyses (PCA) generated using METABOANALYST, a comprehensive web-based package for a range of metabolomics applications [67]. 

## 3. Results

### 3.1. Light Intensity Determines Mycorrhizal Root Colonization and Vesicle Abundance

**Figure 2:**
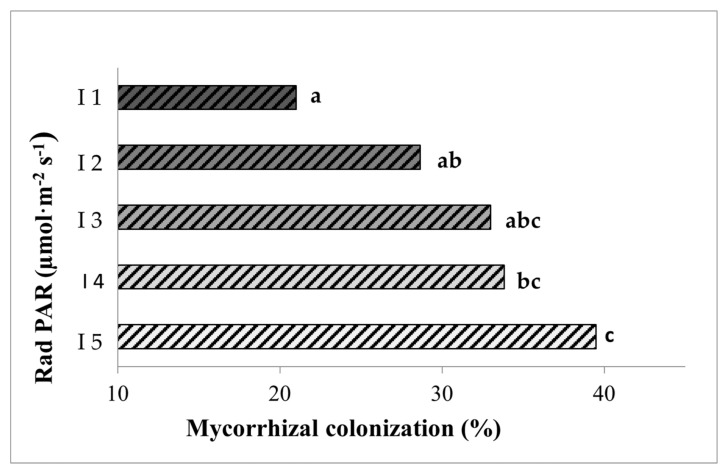
Percentages of root length colonized by the mycorrhizal fungus *Rhizoglomus irregulare* in inoculated lettuce plants grown for seven weeks under the different light intensities. I1 to I5 correspond to 28, 44, 63, 81, and 101 PARs (µmol m^−2^s^−1^), respectively. Data not sharing a letter in common are statistically different according to the LSD test (*p* < 0.05, *n* = 6).

We tested if mycorrhizal colonization is dependent on light intensity by comparing mycorrhizal colonization level in plants grown under five different light intensities, ranging from a very low intensity (I1, 28 PAR) up to a high intensity (I5, 101 PAR). Our results show a clear positive effect of increasing light intensity on root colonization, since values of total root length colonized ranged from 21% for the lower intensity (I1) to almost 40% for the higher intensity (I5) (Figure 2). 

**Figure 3:**
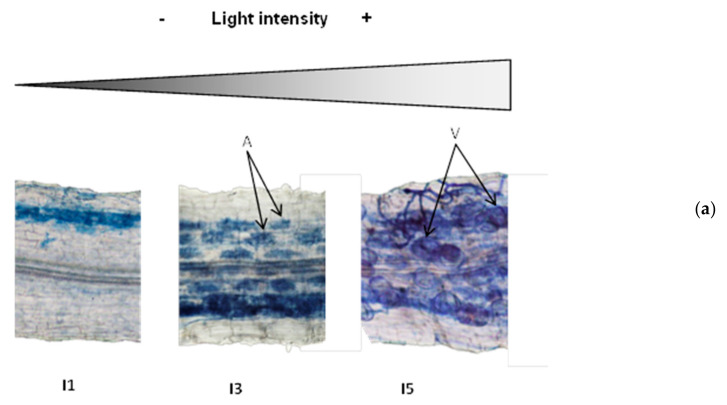

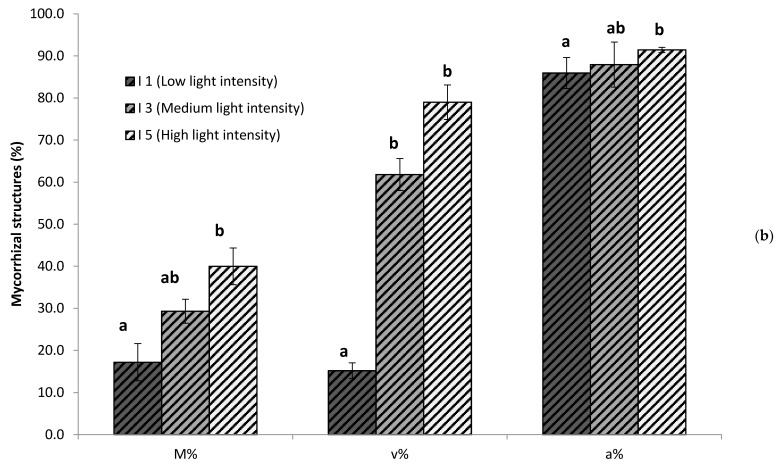
Mycorrhizal colonization and proportions of fungal structures in the root systems of plants grown under different light intensities. (**a**) Representative pictures of fungal colonization within the roots. Arrows illustrate arbuscules (A) and vesicles (V). (**b**) Quantification of mycorrhizal colonization intensity of the root system (M%) and abundande of vesicles (v%) and abundance (a%) of within the colonized areas,, determined as described by Trouvelot et al. [66]. Values were determined in plants growing under low (I1, 28 µmol m^−2^s^−1^), medium (63 µmol m^−2^s^−1^), and high (I5, 101 µmol m^−2^s^−1^) light intensity. Data not sharing a letter in common are statistically different according to LSD multiple ranged tests (*p* < 0.05, *n* = 6).

For a more detailed analysis of mycorrhizal colonization, we chose to focus on three levels of light intensity: low (I1), medium (I3), and high (I5), since there were no significant differences between light levels I2, I3 and I4. As shown in Figure 3, changes in light intensity resulted not only in quantitative, but also qualitative differences in mycorrhizal colonization. The intensity of root colonization by the mycorrhizal fungus was limited in the low light intensity I1, but was more spread along the cortex for higher light intensities (I3; I5). This positive correlation between light intensity and colonization intensity was confirmed by a detailed mycorrhizal assessment following the Trouvelot method [66] that evaluates not only presence or absence of the fungus, but how densely colonized the root cortex is (M%, Figure 3B). Remarkably, important differences were also found regarding the fungal structures within the roots. Several fungal structures are present in mycorrhizal roots, including hyphae (intercellular or intracellular), arbuscules, and vesicles. Arbuscules are the most specific structures of this type of symbiosis, where highly branched intracellular fungal hyphae, surrounded by the cell plasma membrane, greatly increase the surface of nutrient exchange between the plant and the associated fungi (Figure 3A, arrows, A). While all areas colonized by the fungus showed arbuscules and vesicles, the fungal reservoir structures (Figure 3A, arrows, V) were almost absent under low light intensities, but their presence strongly increased with light intensity (Figure 3A,B). The proportions of arbuscules within the colonized areas were similar for the different light intensities (around 90%), whereas the presence of vesicles clearly related to the light intensity, ranging from 15% in I1 to up to 80% in I5 (Figure 3B).

### 3.2. Light Intensity Impacts Photosynthesis, Plant Biomass and Mycorrhizal Growth Response

As expected, photosynthesis increased with increasing light intensity, although this increase was only significant in mycorrhizal plants (Table 2). Plant biomass also increased with light intensity, whereas hypocotyl elongation was inhibited (Table 2). Multifactorial ANOVA confirmed a very significant impact of the light factor (*p* < 0.000), but also significant impacts of mycorrhizal colonization (*p* = 0.008) and their interaction (*p* = 0.003) on plant growth parameters. Under low light intensities, a slight, not significant increase in shoot and root biomass was observed in mycorrhizal plants. Strikingly, a negative mycorrhizal growth response (MGR) was found for both roots and shoots under the higher light intensities, corresponding to the treatments with higher mycorrhizal colonization and higher vesicles abundance (Table 2, Figure 3). 

### 3.3. Light Intensity and Mycorrhizal Colonization Alters Plant Nutrient Contents

**Figure 4:**
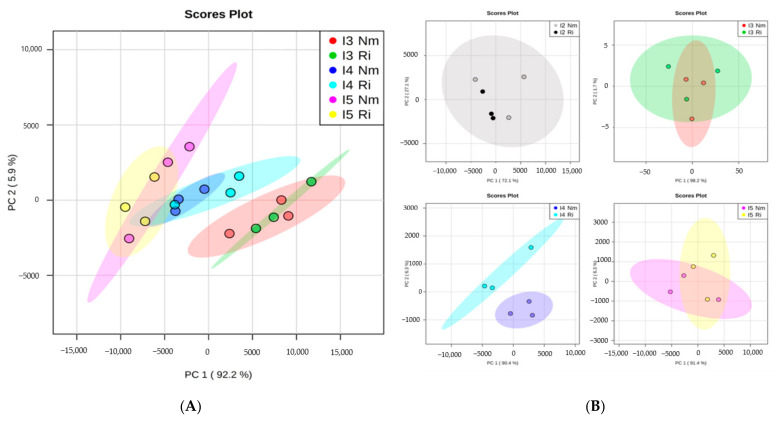
Overview of nutrient organization patterns in lettuce shoots from non-mycorrhizal (Nm) or *Rhizoglomus irregulare*-colonized plants (Ri) grown under different light intensities. I2 to I5 correspond to 63, 81, and 101 PARs (µmol m^−2^s^−1^). (**A**) Principal component analysis (PCA) of nutrients content at different light intensities (I2, I3, I4, I5). (**B**) PCA comparing Nm and Ri for each light intensity level.

To evaluate the impacts of light intensity and mycorrhizal establishment on plant nutrient uptake, the concentrations of mineral nutrients in plants grown under the different treatments were analyzed (Table 3). Multifactorial ANOVA revealed that light significantly altered P, Ca, Cu, K, Mg, Na, and S contents, and mycorrhizal colonization significantly increased P, Na, S, and Zn (multifactorial ANOVA, *p* < 0.05). The interactions of both light and AMF factors were significant only for Mg and Na, and marginally for P (*p* = 0.051) (Table 4). Principal component analysis (PCA) representation allows a general overview of the mineral composition patterns under our different experimental conditions (Figure 4A). Unfortunately, I1 plants were very small, and only two pooled samples were analyzed, so this light intensity was not included in this global statistical analysis. The PCA shows clear impacts of light intensity on the leaves’ mineral element accumulations, while the mycorrhizal treatment had a low general impact with generally overlapping profiles between nonmycorrhizal (Nm) and *R. irregulare*-colonized (Ri) plants for a given light intensity (Figure 4A). However, a more detailed analysis by comparing non-mycorrhizal and mycorrhizal plants at each light intensity separately revealed that changes associated with mycorrhizal colonization were more pronounced at I4 (50% of total mineral content altered), whereas a few mineral changes were detected for the other light intensities (no more than 12.5% of the total mineral content) (Figure 4B). The differences between the profiles of the Nm and Ri plants at I4 are given by significant increases in P, Fe, Cu, Na, S, and Mg in mycorrhizal plants (Table 3). 

### 3.4. Light Intensity Impacts Plant Susceptibility to Botrytis cinerea and Mycorrhiza-Induced Resistance

**Figure 5:**
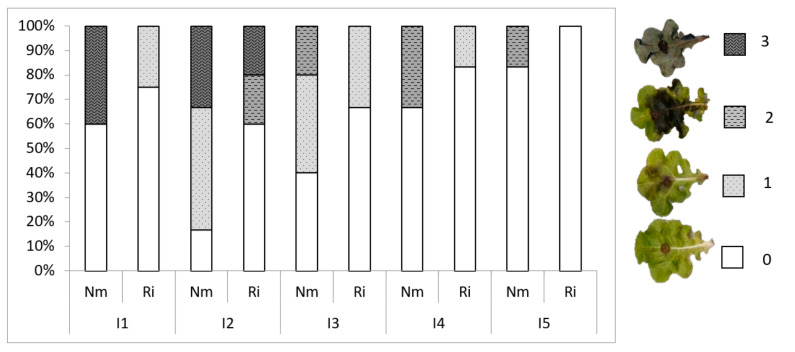
Disease symptoms in mycorrhizal (Ri) or non-mycorrhizal (Nm) lettuce plants grown under different light conditions. Percentage of leaves in the different disease categories for each treatment. Disease index scale: 0, no visible symptoms; 1, necrotic lesions extending below 25% of the leaf surface; 2, necrosis extending more than 50% of the leaf surface; 3: dead leaf.

To explore the effects of light and mycorrhizal colonization on lettuce resistance to foliar pathogens, we performed leaf bioassays with the necrotrophic fungal pathogen *Botrytis cinerea*, the causal agent of grey mold (Table 5). In general, except for I1, the incidence of the disease decreased with light intensity, the percentage of spreading lesions being 67% at I2 and only 17% in I5 for non-mycorrhizal plants. Thus, light intensity impacts basal resistance to *Botrytis cinerea*. Remarkably, disease incidence was lower in mycorrhizal plants at all light intensities, showing a reduction in disease incidence compared to the Nm plants between 38% at I1 and up to 50% or 100% at the highest intensities (I4 and I5, respectively). Moreover, we evaluated the disease development by scoring disease severity in the leaves according to the disease symptoms scale shown in Figure 5. As for incidence, disease severity was reduced by increasing light intensity, the plants being grown under low light very severely damaged, with approximately 40% of leaves showing the most severe necrosis. Plants grown under high light intensities did not reach the most severe damage levels (Figure 5). As for disease incidence, mycorrhizal colonization also reduced *B. cinerea* damage at all light intensities. Noteworthily, mycorrhizal plants did not present leaves in the most severe damage categories at any light intensity, and none of the leaves showed disease symptoms at the highest light intensity (Figure 5).

## 4. Discussion

Light is essential for life, and the impacts of light intensity and quality on plant performance and production are well established. For years, light conditions have been manipulated to improve plant yield and/or quality. The use of microbial inoculants is also a growing trend in modern agriculture, aiming at more sustainable crop management systems, and beneficial fungi have proved their efficiency in improving plant health in different systems [4,7]. Exploring and improving the compatibility between inoculations with beneficial microbes and agriculture practices are major goals of modern agriculture research [68]. In this study, we analyzed the impact of light intensity on mycorrhiza establishment in lettuce plants, and its impacts on plant nutrition, growth, and resistance to pathogen infection. *Rhizoglomus irregulare* is one of the most widespread AMF in nature, and it is used worldwide in commercial mycorrhizal products. It is characterized by its high colonizing ability and the production within the roots of high numbers of vesicles, the AM fungal reservoir structures that, in this species, may eventually became new spores. 

As expected, our results showed that root colonization by *R. irregulare* positively correlated with light intensity. As higher light intensity increases photosynthesis, more resources can be allocated to roots and promote mycorrhizal establishment. In contrast, with declining light intensity, plants should allocate resources to aboveground structures and invest less in mycorrhizas [69,70]. Indeed, several studies have shown that declining light reduces carbon allocation to the fungus and mycorrhizal colonization [34,35,50,51], and reduced sporulation in fungal cultures was observed under shading [71]. 

Besides the extension of mycorrhizal colonization along the root system, the intensity of colonization in the cortex and vesicle abundance strongly increased with light. In contrast to vesicles, arbuscules showed high relative abundance at all light intensities. Thus, functional symbiosis was established at all light levels. Since arbuscules are the main structures for nutrient exchange, we explored the potential impacts of mycorrhizal colonization on plant nutrition and growth. Several growth parameters were determined, and despite a small increase at I2 (although not statistically significant), mycorrhizal colonization had no impact on (I1; I3) or even reduced plant growth at high light intensities (I4; I5). This result is in contrast with previous reports showing significantly enhanced plant growth in mycorrhizal lettuce ([18,56], but differences in MGR have been shown among different plant and fungal genotypes and conditions [57,72]. Regarding the effect of light on MGR, we hypothesized that growth promotion would be observed under high light intensities, as carbon should not be limiting under those conditions. In contrast to our expectations, growth was reduced in mycorrhizal plants under the higher light intensities, likely due to the strong increase in mycorrhizal colonization and the increased formation of lipid containing fungal vesicles. This negative impact on plant growth could be associated with the high carbon demand to support the higher colonization levels and vesicle formation observed under high light conditions. Vesicles constitute an important sink for plant derived carbon [73]. In our study, vesicles were almost absent in roots of plants grown under low light intensities, but their number increased steadily with light, reaching a frequency of 80% in the colonized areas at the highest light intensity level. 

Mycorrhizal symbiosis is known to increase P acquisition, but also other nutrients such as N or Fe, so that improved nutritional value of fruits upon mycorrhization have been reported, for example in tomato [65]. Changes in the nutrient (Fe, Cu, K, Mg, Mn, and Zn) contents of lettuce by mycorrhiza have previously been reported, showing improved nutritional value [58]. Here we showed significant overall increases in P, Mn, S, and Zn in mycorrhizal plants, although light had in general a stronger impact on nutrient contents than mycorrhiza. Nevertheless, the effects of mycorrhiza were more pronounced at the I4 light intensity (81 PAR), where P, Fe, Ni, Cr, Na, and Mg were significantly higher in mycorrhizal plants. All those nutrients are of interest in human nutrition. Thus, nutritional benefits were detected even in the absence of growth promotion effects. Our results indicate that under our experimental conditions, the costs of the high colonization rates and vesicle formation seem to override the photosynthetic and nutritional benefits of the symbiosis. While growth promotion is a common feature of mycorrhizal symbiosis, growth depression has also been reported even within the same plant–AMF combination, and these contrasting mycorrhizal growth responses commonly depend on environmental conditions [33,34,35]. In agreement with this, our results support that the effects of mycorrhizal inoculation on plant growth and nutrient acquisition depend on light availability.

The attention to mycorrhizal symbiosis has been usually devoted to plant growth responses: the benefits of the symbiosis and discussions on a potential continuum between mutualism and parasitism in the interaction have been based merely on these growth benefits [13,74]. However, it is difficult to accept that such widespread symbiosis has survived throughout evolution for more than 400 million years, considering the potential costs and negative effects on plant growth. Other benefits, such as promoting stress resistance, are likely to be of great importance to the plant [4,16,75]. Improved stress resistance in mycorrhizal plants has been amply demonstrated in multiple systems [75]. 

In lettuce, enhanced tolerance to abiotic stress by mycorrhizas has been reported [18,59]. However, the effects of mycorrhizas on lettuce tolerance and resistance to foliar pathogens remain unexplored. We tested here whether mycorrhiza induces resistance against *Botrytis cinerea* infection in lettuce leaves, and the potential influence of light intensity. Light is a regulator of *Botrytis* itself [46,47]. To exclude a potential direct effect of light on *Botrytis* development or infection, the pathogen bioassays were performed in darkness. Therefore, the effect observed should have been derived only from the defensive capacity of the plants (Nm or Ri) grown under the different light regimes. Our results show that susceptibility to *Botrytis* was strongly affected by the light intensity in which plants were grown. Disease incidence and severity decreased with light intensity. Hence, our results evidence a very clear effect of light intensity on the plant’s ability to defend against pathogens, in agreement with the reported light-mediated regulation of jasmonic acid-dependent defenses [40]. Although all (mycorrhizal and non-mycorrhizal plants) followed this pattern of reduced disease incidence and severity under increasing light intensities, mycorrhizal plants always showed lower incidence and symptoms than non-mycorrhizal plants for all light intensities. Thus, mycorrhiza-induced resistance was operative at all light intensities, regardless of the mycorrhizal effect on growth. Previous studies also showed that MIR against *B. cinerea* can still be operative under some nutrient deficiencies [55]. Not only light intensity, but light quality and spectral composition may affect plant defenses and resistance to aggressors [42,76,77], and exploring how these other aspects influence mycorrhizal interactions and MIR will be an exciting field of research. These studies should include analysis of the molecular mechanisms involved, likely involving modulation of plant defenses by phytohormone signaling [77]. In tomatoes, MIR against *B. cinerea* relies on primed defense responses upon pathogen attacks, including JA-dependent defenses and enhanced callose deposition [25,26], and both processes are known to be strongly influenced by light [40,77,78].

Our results highlight that mycorrhizal symbiosis can have important benefits for the host plant that may be unnoticed in studies under controlled conditions focusing only on plant growth or nutritional parameters. Under variable field conditions, plants will likely face different stress situations, and stress resistance benefits may then result in improved plant growth.

In summary, our results evidence that, in addition to its regulatory role in plant nutrition and pathogen resistance, light is a key regulator of mycorrhizal colonization and modulates the symbiosis benefits. Mycorrhization’s effects on lettuce nutrition and growth varied with the light conditions; however, mycorrhizal colonization consistently increased resistance to the foliar pathogen *B. cinerea* plants at all light intensities, regardless of the symbiosis effects on plant growth. The results evidence that mycorrhizal symbiosis’s benefits go beyond host nutrition and growth promotion, and the results can increase our knowledge on the multifaceted effects of mycorrhiza under varying conditions, which is essential for their applications in agriculture.

## Figures and Tables

**Table 1:** Characteristics of the LED tubes.

Code	Power/Consumption	Voltage	Angle of Illuminated Beam	Power Factor	Measurements	Luminous Flux	Color
120018B	18 W	100–250 V	220^o^	>0.90	1200 × 22.5 mm	1500 lm	6500 k.

**Table 2:** Photosynthetic rate and plant growth under the different light intensities. Photosynthetic rate, root and shoot biomass, and hypocotyl length from lettuce plants grown under the different light intensities. I1 to I5 correspond to 28, 44, 63, 81, and 101 PARs (µmol m^−2^s^−1^), and were non-colonized (Nm) or colonized by the arbuscular mycorrhizal fungus *Rhizoglomus irregulare* (Ri). Data are mean values (*n* = 6). Data not sharing a letter in common differ significantly according to ANOVA and Fisher LSD test (*p* < 0.05, *n* = 6).

Light	AMF	Fotosyntetic Rate		FW (g)				DW (g)				Hypocotyl Length (cm)	
		(µmol CO_2_m^−2^s^−1^)		Root		Shoot		Root		Shoot			
**I 1**	**Nm**	0.94	ab	0.3	a	1.9	a	0.02	a	0.07	a	2.0	c
	**Ri**	0.60	a	0.3	a	1.7	a	0.03	ab	0.06	a	1.9	c
**I 2**	**Nm**	1.40	bc	1.1	b	2.9	ab	0.07	abc	0.11	ab	1.7	bc
	**Ri**	1.20	abc	1.4	b	3.1	b	0.11	cd	0.13	bc	1.6	bc
**I 3**	Nm	1.41	bc	1.5	b	4.6	c	0.08	bcd	0,.8	cd	1.2	ab
	**Ri**	1.74	cde	1.5	b	3.9	bc	0.09	bcd	0.15	bc	0.8	a
**I 4**	**Nm**	1.65	cd	3.5	d	6.6	d	0.2	e	0.27	e	1.0	a
	**Ri**	2,.9	e	2.5	c	5.0	c	0.13	d	0.21	d	0.8	a
**I 5**	**Nm**	1.72	cde	3.5	d	6.3	d	0.29	f	0.38	f	0.9	a
	**Ri**	2.22	de	2.6	c	4.8	c	0.2	e	0.3	e	0.9	a

**Table 3:** Mineral nutrient contents in non-mycorrhizal (Nm) and mycorrhizal (Ri) lettuce grown under the different light intensities. I1 to I5 correspond to 28, 44, 63, 81, and 101 PARs (µmol m^−2^s^−1^). Data not sharing a letter in common differ significantly according to ANOVA and Fisher LSD test (*p* < 0.05, *n* = 6).

	I 1		I 2		I 3		I 4		I 5	
	I1Nm	I1Ri	I2Nm	I2Ri	I3Nm	I3Ri	I4Nm	I4Ri	I5Nm	I5Ri
**P**	2370.8 b	2614.1 b	2176.6 b	2514.7 b	2256.7 b	2229.1 b	1528 a	2380.9 b	1505.3 a	1571.5 a
**Ca**	22,832 bc	23,296 c	22,612 c	20,006 a	22,837 c	22,793 c	20,211 ab	19,781 a	19,095 a	19,473 a
**Cu**	12.4 abcd	11 ab	14.7 d	13.7 bcd	11.6 abc	13.6 bcd	11 a	13.8 cd	10.9 a	12 abc
**Fe**	683 abc	536 ab	614 ab	671 abc	627 ab	702 abc	408 a	973 c	858 bc	848 bc
**K**	61,514 f	58,983 ef	54,200 cde	56,190 def	57,277 def	58,963 ef	48,741 abc	51,876 bcd	46,490 ab	43,831 a
**Mg**	5482 cd	5958 d	5534 cd	5190 bc	5471 c	5535 cd	4767 ab	5378 c	4747 a	4785 ab
**Mn**	92.9 c	65.7 a	86.7 c	69.9 ab	89.8 c	81 bc	73.3 ab	73.9 ab	87.9 c	69.7 ab
**Mo**	1.59 ab	1.66 ab	1.71 b	1.26 a	1.45 ab	1.35 ab	1.5 ab	1.48 ab	1.23 a	1.31 a
**Na**	12,556 f	13,947 g	10,937 cd	10,697 bc	11,890 ef	11,743 def	9879 b	11,155 cde	8070 a	8806 a
**Ni**	4.8 c	4.7 c	3.3 b	3.2 ab	2.9 ab	3.7 b	2.4 a	3.6 b	3.6 b	3.1 ab
**S**	4028 c	4050 c	3844 bc	3750 bc	3923 bc	4009 c	3660 b	4009 c	3357 a	3827 bc
**Si**	1241 ab	1166 ab	1228 ab	1007 ab	1043 ab	1305 b	894 a	1074 ab	1235 ab	1239 ab
**Zn**	144 ab	196 c	165 abc	174 bc	155 ab	165 bc	162 ab	167 bc	130 a	144 ab

**Table 4:** *p*-values for each nutrient according to two-way ANOVA on the main effects of light intensity (Light), mycorrhizal colonization (AM), and their interaction (Light x AM). Significant values (*p* < 0.05) are in bold.

*P* Value	P	Ca	Cu	Fe	K	Mg	Mn	Mo	Na	Ni	S	Si	Zn
**Light**	**0.000**	**0.001**	**0.035**	0.309	**0.000**	**0.000**	0.130	0.204	**0.000**	**0.001**	**0.004**	0.367	0.059
**AMF**	**0.006**	0.407	0.212	0.107	0.800	0.098	**0.000**	**0.036**	**0.008**	0.190	**0.018**	0.730	**0.024**
**L x AMF**	0.051	0.346	0.105	0.072	0.473	0.037	0.059	0.319	0.050	0.053	0.052	0.384	0.420

**Table 5:** Disease incidence of *Botrytis cinerea* infection in leaves of non-mycorrhizal (Nm) or mycorrhizal (Ri) lettuce plants grown under different light conditions. (I1 to I5 correspond to 28, 44, 63, 81, and 101 PARs (µmol m^−2^s^−1^)). Leaves were inoculated with *B. cinerea* and incubated under high humidity and darkness for 6 days. The disease incidence is expressed as the percentage of leaves in each treatment showing developing necrotic lesions. Mycorrhiza-induced resistance (MIR) is calculated as the reduction in disease incidence observed in Ri plants when compared to Nm plants for each light intensity.

Treatment		Disease	MIR
		Incidence (%)	
I1	Nm	40	38%
	Ri	25	
I2	Nm	67	40%
	Ri	40	
I3	Nm	60	45%
	Ri	33	
I4	Nm	33	50%
	Ri	17	
I5	Nm	17	100%
	Ri	0	

## Data Availability

All data is available in the manuscript.
